# Evidence for history-dependence of influenza pandemic emergence

**DOI:** 10.1038/srep43623

**Published:** 2017-03-02

**Authors:** Edward M. Hill, Michael J. Tildesley, Thomas House

**Affiliations:** 1 Centre for Complexity Science, University of Warwick, Coventry, CV4 7AL, United Kingdom; 2 Zeeman Institute: Systems Biology and Infectious Disease Epidemiology Research (SBIDER), University of Warwick, Coventry, CV4 7AL, United Kingdom; 3 School of Life Sciences, University of Warwick, Coventry, CV4 7AL, United Kingdom; 4 Mathematics Institute, University of Warwick, Coventry, CV4 7AL, United Kingdom; 5 School of Mathematics, The University of Manchester, Manchester, M13 9PL, United Kingdom

## Abstract

Influenza A viruses have caused a number of global pandemics, with considerable mortality in humans. Here, we analyse the time periods between influenza pandemics since 1700 under different assumptions to determine whether the emergence of new pandemic strains is a memoryless or history-dependent process. Bayesian model selection between exponential and gamma distributions for these time periods gives support to the hypothesis of history-dependence under eight out of nine sets of modelling assumptions. Using the fitted parameters to make predictions shows a high level of variability in the modelled number of pandemics from 2010–2110. The approach we take here relies on limited data, so is uncertain, but it provides cheap, safe and direct evidence relating to pandemic emergence, a field where indirect measurements are often made at great risk and cost.

Influenza A is a cause of considerable morbidity and mortality in humans. Occasionally, humans become infected with a strain of influenza derived from non-human sources, which are essentially novel to humans. This can give rise to a localised outbreak that may develop into a worldwide influenza pandemic^1^. Though efforts are being made to improve pandemic influenza risk assessment, it is currently not possible to predict which non-human influenza A virus will cause the next pandemic or when it will emerge^2^,^3^.

Identifying possible causal (biological) mechanisms for emergence of strains with pandemic potential often relies on indirect measurements from laboratory and field surveillance studies, such as viral sequencing of archaic pathogens^4^,^5^ and seroprevalence analysis^6^, which are undertaken at great risk and cost. Furthermore, recent experimental studies setting out to determine the necessary mutations for specific influenza strains to become transmissible among mammals^7^,^8^,^9^ have been controversial^10^. Thus, we argue that adopting a mathematical modelling approach can potentially provide cheap, safe and direct validation of proposed assumptions regarding pandemic emergence. This may subsequently help inform the type of interventions that would have the greatest impact in reducing the risk of further influenza pandemic occurrences.

When referring to influenza, a pandemic signifies a world-wide epidemic caused by an emergent influenza A strain that transmits among humans, was not previously circulating among humans and to which most people do not have immunity^11^. Therefore, only virological techniques allow recognition of a pandemic with certainty. Nevertheless, work on early influenza outbreaks has argued that if the epidemic originated in one place, and from there spread world-wide with high morbidity, it was likely to have been a pandemic^12^,^13^,^14^,^15^. This allows a history of presumptive influenza pandemics since 1700 to be constructed, along with the waiting times between pandemic outbreaks.

From this, we can analyse statistically which of two opposing mechanisms can plausibly generate the waiting time data; a memoryless process or a history-dependent process. For the memoryless process, the time to the next pandemic is not influenced by how much time has passed since the previous pandemic. Outbreaks of Ebola are thought to follow such a process and are typically modelled under this assumption^16^. On the other hand, in a history-dependent process, the probability of an event occurring is influenced by the elapsed time since the last such event. Such a process may feasibly arise through a combination of contributing mechanisms. First, a currently circulating strain may need to undergo a required accumulation of mutations to develop pandemic potential, as found for specific H5N1 influenza strains requiring only a small number of mutations to become transmissible among mammals such as ferrets^7^,^8^. Second, as a result of population immunity to prior strains, as suggested by the recycling theory for pandemic emergence^17^,^18^. The recycling theory hypothesises that influenza A viruses with similar or identical hemagglutinins can re-emerge over time, with only a significant proportion of then-living individuals aged above a particular threshold age immunologically protected from the emergent virus. In effect, the spacing between pandemics is caused by waning population-level immunity to a previous pandemic strain, with the proportion of the population immunologically protected diminished by both age-related mortality and the influx of immunologically naive newborns.

Here, we utilise waiting times between presumptive influenza pandemics since 1700 and ascertain via a Bayesian analysis whether they are better modelled by a memoryless or history-dependent process. Using prior information gathered from hypotheses related to the emergence of flu strains, we demonstrate from this small but informative dataset evidence that spillover of strains with pandemic potential is likely to be a history-dependent process. With a weaker assumption of uninformed priors, conclusions may only be inferred if we are sure about the legitimacy of the contested pandemics. Forward simulations using the preferred models are then performed to obtain predictions for the number of influenza pandemics we are likely to see in the 100 years following the last pandemic.

## Methods

### Historic pandemic influenza data

We considered possible pandemics from 1700 to the present. Three historic pandemic timelines were constructed based on the number of supporting sources for each epidemic being a pandemic. Between-pandemic waiting times were obtained for each timeline.

All historic pandemic timelines proposed here contained at least four post-1900 pandemics, beginning in 1918, 1957, 1968 and 2009 respectively. In addition, seroarcheological observations have compellingly linked the emergence of an H3 influenza virus to a pandemic beginning in 1889^18^. Thus, we included 1889 in all three timelines. Note there is a reputed influenza pandemic stated to have begun around 1900^12^,^14^. However, this epidemic was thought to have occasioned the emergence of the H3 pandemic virus, which was later found to have emerged in the preceding 1889 pandemic as just described^18^. In light of these findings this outbreak is not universally considered a genuine pandemic, hence we did not include it within our analysis.

For the period 1700 up to 1889, recorded influenza epidemics that may have satisfied the criteria to be classed as a pandemic were obtained from Patterson^12^, Beveridge^13^ or Taubenberger and Morens^15^. These particular data sources were selected due to each providing a varied account of putative influenza pandemics since 1700, while collectively representing a range of possible occurrences.

‘Timeline’ A had the most stringent inclusion criteria. Along with the pandemics that have occurred from 1889 onwards, it only included additional epidemics during 1700–1888 agreed on across the multiple sources as being pandemics. However, we had no waiting time information for the pandemic in 1729, which left us with seven waiting times. For ‘Timeline B’, an additional five possible pandemics were included that were given in either Beveridge^13^ or Taubenberger and Morens^15^. Our most inclusive case, labelled ‘Timeline C’, included 1732 as a separate pandemic as it is given as being distinct from the 1729 pandemic by some of the earlier sources^12^,^13^, though it is not known whether these were two pandemics separated by a very short interval or one pandemic with a long-delayed recurrence^15^. Furthermore, this timeline also included the 1977 re-emergence of H1N1, which is widely believed to have occurred due to human factors rather than the biological processes that are the main interest here^19^,^20^. Note that although only the post-1900 pandemics have been virologically confirmed, efforts to draw useful conclusions would be hindered by the size of a dataset comprising only those pandemics, primarily due to the penalisation of the additional complexity in the history-dependent model when there are few observations. As a consequence, we did not analyse such a scenario here. Complete lists of each pandemic timeline are provided in Table 1.

### Pandemic influenza emergence - model fitting and comparison

Our investigation into the plausibility of the observed waiting time data being generated via a memoryless or history-dependent process was formulated as a model selection problem between exponential and gamma distributions. Strictly speaking, if we believe that history dependence is generated by a combination of several memoryless events, then we should use a very general class of distributions called *phase-type*, which in fact can provide an arbitrarily good approximation to almost any probability distribution^21^,^22^. In practice, however, any phase-type distribution that arises from a realistic combination of events will be very close to a gamma distribution and we therefore consider only gamma distributions as representations of history dependence, while noting that these may not be adequate if an unexpectedly complex set of individually memoryless events drives pandemic emergence.

**Figure 1:**
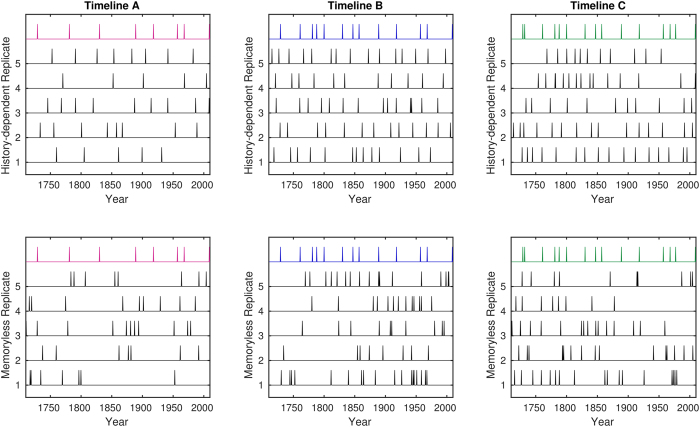
Visualisation of the observed timeline data and models. Timeline data is shown as a coloured series at the top of each plot, above five replicates for the fitted history-dependent (top row) and memoryless (bottom row) models.

While visual comparison of the three influenza pandemic timelines versus simulations from fitted model parameters suggest that the memoryless realisations have more clusters and long gaps than the real data (Fig. 1), human assessment of randomness is notoriously poor^23^. We therefore note the importance of performing a formal statistical analysis on the available data. Owing to the small size of the data, we performed a Bayesian analysis using reversible jump Markov chain Monte Carlo (RJMCMC)^24^. In this framework the strength of evidence for each hypothesis is given by a probability. While a typical convention in Bayesian model selection is to use Bayes factors to qualitatively categorise the evidence against a null hypothesis^25^, we instead deal directly with the marginal likelihoods due to the small nature of the datasets considered. These marginal likelihoods correspond to the rational subjective probabilities for each hypothesis. Such an approach is more appropriate than simply selecting one model over another when faced with a small but informative dataset.

While both exponential and gamma distributions incorporate a rate parameter, *λ*, the gamma distribution has an additional parameter *κ*, the shape parameter. This can be interpreted as a proxy for a specified number of events needing to occur before the next pandemic outbreak can begin. For the rate parameter, an uninformative Uniform(10^−3^, 1) prior was selected, representing a prior belief that pandemics are neither annual nor extremely infrequent. For *κ*, we considered three different priors containing different amounts of information about the mechanism behind pandemic emergence. The first of these assumed that the timing of pandemics was not very regularly spaced, or equivalently that the number of mutations required to cause a pandemic was not particularly large. To describe this, an uninformed ‘not clockwork’ *κ* prior of Uniform(0, 25) was used. This captures a prior belief that new pandemics are not very regular (the level of regularity for different values of *κ* is visualised in Supplementary Fig. S1), while assuming no prior preference for specific values in the range over others. However, findings from experimental studies allow us to suggest alternatives for the prior informed from hypotheses relating to emergence of flu strains (e.g. only four or five mutation steps are needed for specific H5N1 influenza strains to become transmissible between ferrets^7^,^8^). Two differing informed priors for *κ* were selected to incorporate this additional mechanistic information, which had the same mean (four) but dissimilar variability to account for contrasting levels of certainty. The first was a ‘weakly mechanistic’ Exp(1/4) prior, which gives lower prior credibility to very regular pandemics (large values of *κ*) than the uniform prior. The second was a ‘strongly mechanistic’ 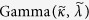 prior, with shape parameter 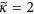 and rate parameter 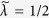, which gave little credibility for both very regular pandemics and for one influenza pandemic being able to immediately follow another (i.e. small values of *κ*). For each of the three choices of prior for *κ* we fit the data for each of the three timelines separately, giving nine different sets of modelling assumptions.

For each dataset the RJMCMC sampler was run until we obtained 10^5^ samples, thinning by a factor of twenty sweeps with a burn-in period of 10^4^ sweeps. Within each sweep within-model moves for each parameter were tried using the Metropolis-Hastings algorithm^26^,^27^, before a trans-dimensional move was attempted. The acceptance rates for these cross-model moves were recorded to track sampler performance. Higher acceptance rates lead to lower autocorrelation in the *k* chain (where *k* denotes the model/hypothesis, with the data following an exponential distribution when *k* = 1, and a gamma distribution when *k* = 2), while near-zero acceptance rates would indicate the sampler was not performing adequately due to being highly inefficient in exploring the parameter space. The implementation of these moves, along with formal model definitions, are described in the Supplementary Methods.

### Pandemic influenza emergence - model suitability

To assess goodness-of-fit Monte Carlo simulations were performed for each of our fitted memoryless and history-dependent models, using 10^4^ samples generated from the RJMCMC procedure. A simulation duration equivalent to 300 years was chosen to approximately match the timespan of our historic pandemic lists. Empirical cumulative distribution functions (ECDF) of the inter-event time between pandemics were computed for each simulation run. These were compared to the observed data through survival functions (1 - ECDF).

### Validation - Ebola outbreak analysis

The validity of the method was checked by carrying out a similar model fitting and comparison procedure on the waiting time data for Ebola outbreaks (the month and year of each outbreak obtained from sources listed by the CDC^28^), with the expectation being the memoryless model would be strongly preferred since Ebola is too virulent in humans to expect to see phenomena such as population-level immunity or long-term evolution. Parameter priors used in this case were Uniform(10^−3^, 1) for *λ* and Uniform(0, 25) for *κ*.

### Forward simulation outline

For each pandemic influenza timeline and prior type combination, 10^4^ samples generated from the RJMCMC procedure were used to simulate forward 100 years (from 2010 to 2110). The number of samples drawn from each model was weighted by the posterior model probabilities for the given timeline. Distributions of the proportion of simulations giving a specified number of influenza pandemic events in this time period were constructed, allowing for between-timeline comparisons. All calculations and simulations were performed with MATLAB^®^.

## Results

### Pandemic influenza emergence - model fitting and comparison

Running our RJMCMC sampler on each timeline assuming an uninformed ‘not clockwork’ *κ* prior gave good levels of between-model mixing, with cross-model jump acceptance rates between 30% and 60%. In contrast, the informed ‘mechanistic’ *κ* prior cases had cross-model jump acceptance rates between 15% and 40%, though the thinning used reduced autocorrelation in the *k* chain to ensure the samples drawn could be considered as independent.

When using the ‘not clockwork’ *κ* prior, the gamma distributed model (history-dependent hypothesis) had higher posterior probabilities for Timelines A and B (0.70 and 0.77 respectively). Nevertheless, for Timeline C the history-dependent hypothesis had less support than the memoryless hypothesis (0.45 vs 0.55). Consequently, this prior assumption only lets us infer something if we are sure which pandemic timeline is correct. On the other hand, using ‘mechanistic’ priors informed by studies related to emergence of flu strains resulted in model probabilities between 0.70 and 0.92 for the history-dependent hypothesis across our range of exclusive to inclusive histories of pandemic influenza (Table 2).

**Figure 2:**
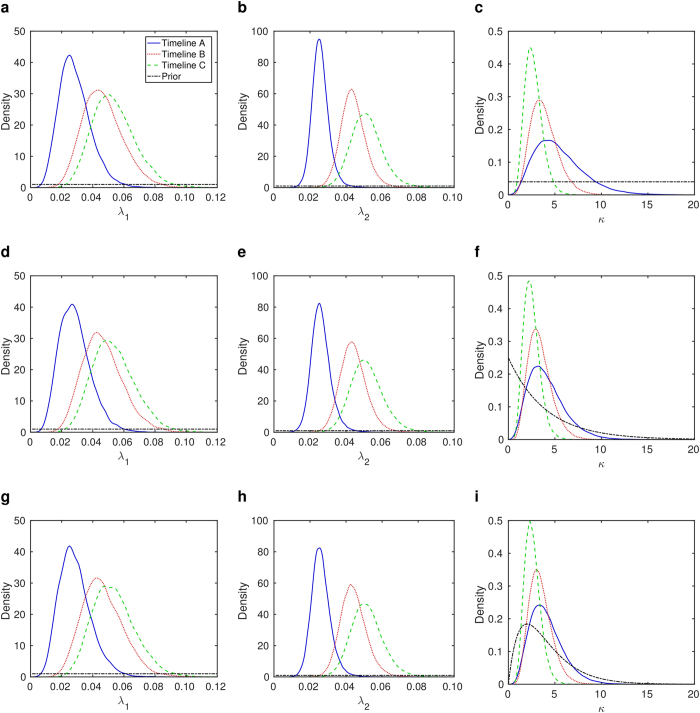
Parameter probability distribution functions estimated from RJMCMC output when using the specified prior for *κ*. (**a**–**c**) Not clockwork; (**d**–**f**) weakly mechanistic; (**g**–**i**) strongly mechanistic. (**a**,**d**,**g**) Density estimate for *λ*_1_ where *k* = 1; (**b**,**e**,**h**) density estimate for *λ*_2_ where *k* = 2; and (**c**,**f**,**i**) density estimate for *κ* where *k* = 2.

Comparing across the different *κ* priors, the median and 95% credible intervals for each model parameter are quantitatively similar. Unsurprisingly, the main exceptions to this were the ‘weakly mechanistic’ and ‘strongly mechanistic’ prior resulting in tighter posterior distributions for *κ* compared to when the ‘not clockwork’ prior was used (Fig. 2 and Supplementary Table S1). Timeline A exhibits the widest posterior distributions for *κ*, along with a greater median value for *κ* and lower *λ* median values. This coincided with a broader distribution for the standard deviation *σ* (Supplementary Table S2), implying longer and greater variability in spacing between influenza pandemics relative to Timelines B and C.

### Pandemic influenza emergence - model suitability

**Figure 3:**
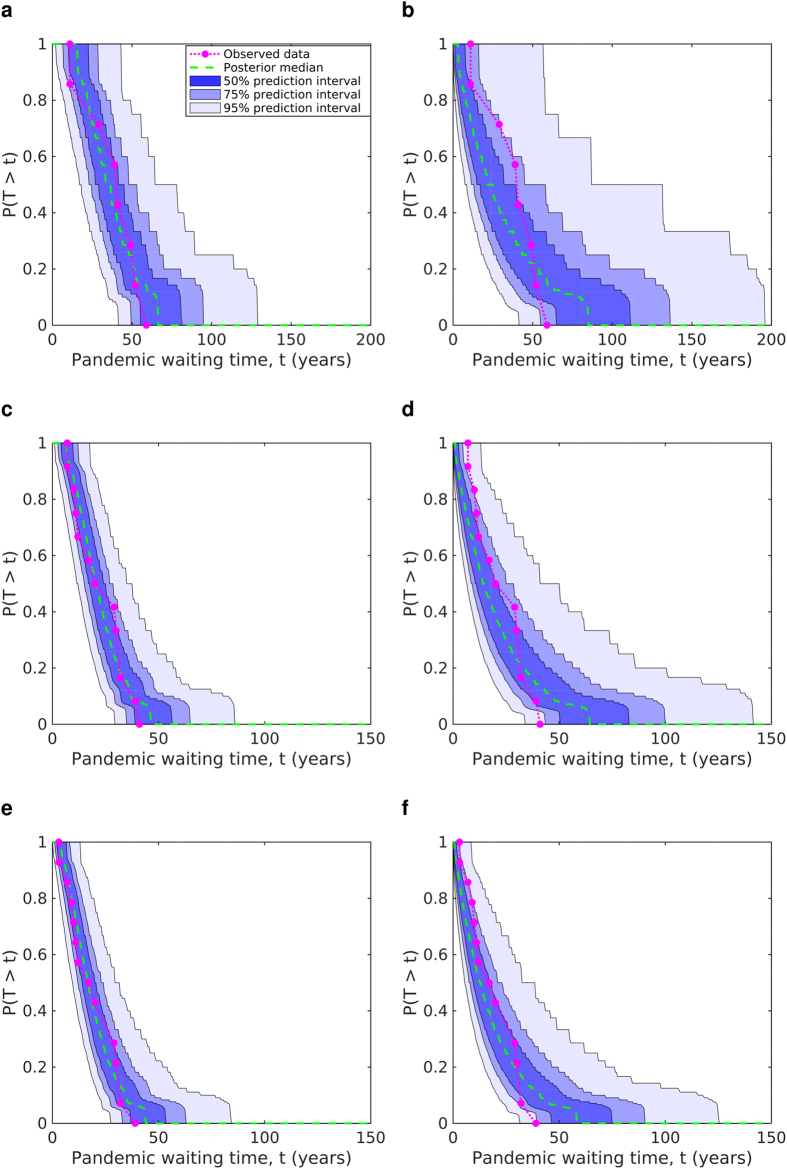
Predicted posterior influenza pandemic inter-event time survival functions versus the empirical survival function, under the ‘strongly mechanistic’ prior assumption. Waiting time model fits relative to the observed data (magenta dotted line) under the following hypothesis: (left) history-dependent; (right) memoryless. Across all proposed historic pandemic lists the history-dependent hypothesis corresponds adequately with the observed data. (**a**,**b**) Timeline A; (**c**,**d**) timeline B; (**e**,**f**) timeline C. Median posterior survival functions are given by the green dashed lines, with prediction intervals of 50%, 75% and 95% represented by shaded blue regions (moving from darkest to lightest).

For each of our nine sets of modelling assumptions the posterior simulated survival function distributions for pandemic event waiting times indicated a reasonable model fit to the data when a history-dependent hypothesis for influenza pandemic emergence was assumed. A good level of agreement was observed in particular under this hypothesis in combination with the ‘strongly mechanistic’ prior assumption for *κ*, with the predicted median waiting time survival function closely tracking the empirical data. On the other hand, the memoryless model exhibited greater inter-event time variability, with the predicted survival function distributions generally declining sooner and having a longer tail relative to the data (Fig. 3). This reaffirms the fact that such an assumption lends greater credibility for both long interludes between pandemics (exceeding 100 years) and for one influenza pandemic being able to immediately follow another relative to what truly occurred. Similar outcomes were obtained using the alternative *κ* prior distributions (Supplementary Figs S2–S3).

### Validation - Ebola outbreak analysis

When fitting our models to the Ebola outbreak waiting times we obtained 10^5^ samples, thinning by a factor of 100 sweeps with a burn-in period of 10^4^ sweeps. As expected, the memoryless model was strongly favoured compared to the history-dependent model, with posterior model probabilities of 0.95 and 0.05 respectively. Furthermore, this weight of evidence was supplemented by the inferred gamma model being exponential like in nature, with the following parameter median and 95% credible intervals obtained: *κ* = 0.719 (0.429, 1.15); *λ*_2_ = 0.618 (0.366, 0.925). Similar values were found for the exponential model rate parameter, *λ*_1_ = 0.619 (0.403, 0.890).

### Forward simulation analysis

**Figure 4:**
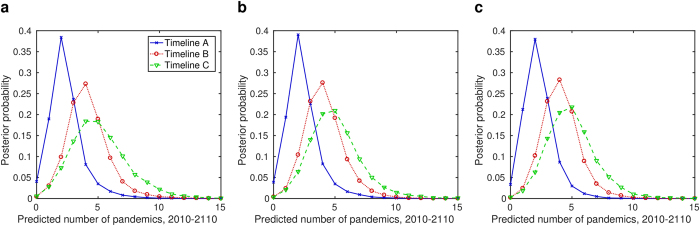
Posterior predictive distributions for the number of pandemics between 2010–2110, for each timeline and choice of *κ* prior. (**a**) ‘Not clockwork’ *κ* prior case; (**b**) ‘weakly mechanistic’ *κ* prior case; (**c**) ‘strongly mechanistic’ *κ* prior case.

Across our range of inclusivity criteria for proposed pandemics, the lowest mode for the number of predicted pandemics during 2010–2110 was obtained by Timeline A (two). In contrast, Timelines B and C gave modes of four or five (Fig. 4). Further, Timeline A displayed less variability in the predicted number of pandemic events, with the majority of simulations predicting two or fewer pandemics between 2010–2110. When the ‘not clockwork’ *κ* prior was used, the proportion of simulations predicting two or fewer pandemics was 62% for Timeline A, compared to just 13% and 11% for Timelines B and C respectively. Quantitatively similar results were obtained using either ‘mechanistic’ *κ* prior (for the ‘weakly mechanistic’ prior in particular, Timeline A: 62%, Timeline B: 13%, Timeline C: 8.9%).

Timeline C had a more diffuse distribution than Timeline B, despite including only a couple of additional waiting times. Explicitly, Timeline C attributed a greater weight of support to there being 10 or more pandemics between 2010 and 2110 (4.6% versus 1.1% of simulations). This discrepancy between the predictive pandemic event distributions remained using the ‘mechanistic’ *κ* priors, although these choices of prior did result in reduced absolute probabilities of an extreme number of pandemics (10 or more) occurring in the time period of interest (for the ‘weakly mechanistic’ prior, Timeline B: 0.63%, Timeline C: 3.2%).

## Discussion

Assessing influenza pandemic emergence risk is an ongoing public health concern^2^,^3^. This study was motivated by the view that mathematical approaches could provide cheap ground-truthing and validation of expensive and sometimes hazardous laboratory and field surveillance efforts to understand pandemic emergence. Our findings suggest evidence for the emergence of influenza pandemics being plausibly generated by a history-dependent process, although this does depend on the prior assumptions made. Even with the data being limited, utilising a RJMCMC approach to model selection indicates in a mathematically rigorous sense the strength of evidence for each hypothesis, improving upon merely selecting one model over another. Our analysis of the Ebola outbreak waiting time data, finding the memoryless hypothesis to be strongly preferred, provides confirmation that the method works as expected for another real system.

Under an uninformed ‘not clockwork’ prior assumption, the preferred model mechanism depended on the strictness of the proposed pandemic inclusion criteria, only letting us infer model preference if we are sure which pandemic timeline is correct. We note, however, that advances in the recovery and sequencing of genetic information has led to analyses of ancient specimens, meaning genomic data from archaic pathogens (obtained from corpses) can be gathered. Recent examples include characterisation of the 1918 pandemic influenza virus^4^,^5^ and reconstruction of the bubonic plague genome from victims of the Black Death^29^. Further discovery and sequencing of past influenza viruses may weaken or strengthen the claims of the contested pre-20th century pandemics, culminating in the accuracy of the list of historic influenza pandemics being enhanced.

Nevertheless, using a ‘mechanistic’ prior informed by experimental studies suggests much stronger evidence for predictability of pandemics, with the spillover of strains with pandemic potential from the animal reservoir to humans likely to be a history-dependent process. This would be consistent with strains needing to accumulate specific mutations and predictions of, for example, the recycling theory^17^,^18^, with the history-dependent process arising as a result of population immunity to prior strains. Further support is given by previous studies determining that all the emergent viruses that caused the post-1900 influenza pandemics resembled those that had circulated previously within the lifespan of then-living people^30^. Specifically, the 1968 pandemic virus was similar to the one that caused the 1889 pandemic^17^,^18^, while the H1 2009 pandemic virus was most similar to viruses that circulated before 1947^31^. Such an immunity argument could also explain why there was very strong support for Ebola outbreak inter-event times being memoryless, with each of these outbreaks not being capable of establishing population-wide immunity due to being relatively small and having a high mortality rate^28^. With the majority of the population remaining susceptible, we would expect the likelihood of the next outbreak occurring within a given time (originating from zoonotic transmission events from animal hosts) to be unaffected by the time passed since the preceding outbreak.

In this case, there would be significant merits in having active surveillance in humans and zoonotic hosts to reveal the diversity of influenza strains in circulation and the immunological profile of the population. Current immune landscape studies, such as Fluscape^32^,^33^ and seroprevalence analyses of live bird market workers^6^, provide example frameworks that could be extended to investigate the immune profiles of the population to strains widely circulating in livestock populations. Such knowledge would aid classification of currently circulating strains with respect to their pandemic potential, determining those that pose a considerable threat. Going a step further, earlier detection of strains with pandemic causing capability could allow the containment of the strain to a localised area through, for example, more aggressive culling controls and movement restrictions in farmed poultry. Additionally, active surveillance outputs may inform the composition of influenza virus vaccines to be developed and administered to the at-risk human population as part of pandemic preparedness procedures. However, current influenza virus vaccines have limitations, requiring frequent updates to reflect the antigenic changes that occur in the pool of circulating virus strains. Circumventing this drawback is a key area of ongoing research through the development of ‘universal’ influenza vaccines that are less sensitive to the antigenic evolution of the virus, therefore giving broader protection against emergent strains^34^,^35^.

For all three suggested pandemic influenza histories, forward simulations implied a wide variability in the possible number of expected pandemics in the next 100 years following the last pandemic. Although Timeline A offers greater support for there being two or fewer pandemics between 2010–2110 compared to either Timeline B or C, additional virological information is required to determine which of these timelines is representative of the actual history of pandemic influenza. If this is achieved, the relevant timeline findings can be used to quantify the risk of a set number of pandemic events occurring in the next 100 years. Note that these predictions could be viewed as a worst case scenario, due to not accounting for the impact of future intervention measures. We anticipate however that the implementation of interventions that successfully determine and prevent the spread of strains posing the largest pandemic risk, possibly through the strategies discussed above, will diminish the likelihood of them realising their full pandemic potential. The number of influenza pandemics would drop as a result.

Our modelling framework posed the following potential issues. First, there was data uncertainty as a result of conflicting accounts of what were true historic influenza pandemics within the literature. As a consequence, to account for a range of possible pandemic histories, we constructed our three distinct pandemic timelines with strict, moderate and relaxed inclusion criteria based on the amount of corroboration across sources for an outbreak being a pandemic. Second, result robustness is likely weakened due to the small nature of the dataset and sensitivity with respect to the timeline used. We argue, however, that the observed dates of influenza pandemics are the ultimate test of our understanding of novel strain emergence, and with this data the more complex hypothesis was still found to be preferred when including a varied number of pre-20th century pandemics. Third, for the modelling framework a number of subjective decisions had to made. For our history-dependent model, alternative distributions could have been selected (e.g. Weibull distribution), though due to any phase-type distribution arising from a realistic combination of events being very close to a gamma distribution we only considered gamma distributions as representations of history dependence here. Additionally, other priors for *κ* could be used since, as expected, our prior specification for *κ* impacts on the posterior support for the history-dependent model. Although our ‘strongly mechanistic’ prior may be overly restrictive in assuming low credibility for one influenza pandemic being able to immediately follow another, such assumptions were not made with the ‘weakly mechanistic’ prior. Further, it is noteworthy that the weakly informed prior had no qualitative impact on the findings when compared to the findings using the ‘strongly mechanistic’ *κ* prior.

In summary, our analysis of influenza pandemic waiting times found support for the hypothesis of influenza pandemic emergence being history-dependent, rather than a memoryless process, under eight out of nine sets of modelling assumptions. Although the approach utilised here is reliant on limited and uncertain data, it provides cheap and direct validations of indirect measurements from expensive and sometime dangerous laboratory and field surveillance efforts that aim to further our understanding of pandemic emergence.

## Additional Information

**How to cite this article**: Hill, E. M. *et al*. Evidence for history-dependence of influenza pandemic emergence. *Sci. Rep.* **7**, 43623; doi: 10.1038/srep43623 (2017).

**Publisher's note:** Springer Nature remains neutral with regard to jurisdictional claims in published maps and institutional affiliations.

## Supplementary Material

Supplementary Information

## Figures and Tables

**Table 1:** Complete lists of the pandemics included in each of our proposed pandemic influenza timelines.

Date	Popular Name	Subtype	Timeline A	Timeline B	Timeline C
1729					
1732			—	—	3 years†
1761			—	32 years*	29 years*
1781			52 years	20 years	20 years
1788			—	7 years*	7 years*
1800			—	12 years†	12 years†
1830			49 years	30 years	30 years
1847			—	17 years†	17 years†
1857			—	10 years†	10 years†
1889	Russian Flu	H3N?	59 years	32 years	32 years
1918	Spanish Flu	H1N1	29 years	29 years	29 years
1957	Asian Flu	H2N2	39 years	39 years	39 years
1968	Hong Kong Flu	H3N2	11 years	11 years	11 years
1977	Russian Flu	H1N1	—	—	9 years||
2009	Swine Flu	H1N1	41 years	41 years	32 years

Start date (assumed point of new strain) is given, as well as popular name and most likely subtype. The delay dates for each timeline are given, and pandemics assumed not to have happened in the timeline are denoted —.

^*^Only listed as a probable pandemic by Taubenberger and Morens^15^.

^†^Not listed as a separate and/or probable pandemic by Taubenberger and Morens^15^.

^||^Widely believed to have occurred due to human factors, thus only considered within the most inclusive timeline.

**Table 2:** Posterior probabilities given to the history-dependent hypothesis for each timeline and choice of *κ* prior.

	*κ* prior		
	Not clockwork	Weakly mechanistic	Strongly mechanistic
1. Timeline A	0.70	0.82	0.86
2. Timeline B	0.77	0.89	0.92
3. Timeline C	0.45	0.73	0.77
