# Corrigendum: Evidence for history-dependence of influenza pandemic emergence

**DOI:** 10.1038/srep46770

**Published:** 2017-05-04

**Authors:** Edward M. Hill, Michael J. Tildesley, Thomas House

Scientific Reports 7: Article number: 4362310.1038/srep43623; published online: 03 02 2017; updated: 05 04 2017

This Article contains errors in Reference 11 which was incorrectly given as:

World Health Organisation. Frequently Asked Questions on human infection caused by the avian influenza A(H7N9) virus. URL http://www.who.int/influenza/human_animal_interface/faq_H7N9/en/ (2014).

The correct reference is listed below:

World Health Organisation. Influenza virus infections in humans (February 2014). URL http://www.who.int/influenza/human_animal_interface/virology_laboratories_and_vaccines/influenza_virus_infections_humans_feb14.pdf (2014).

