# Supplementary material for: Evidence for history-dependence of influenza pandemic emergence
Source: Sci Rep. 2017 Mar 2;7:43623. doi: 10.1038/srep43623 (PMC5333635; doi:10.1038/srep43623)
Supplement: Supplementary Information [file srep43623-s1.pdf]

Evidence for history-dependence of influenza pandemic emergence: Supplementary Information

Edward M. Hill Michael J. Tildesley Thomas House

Table of Contents

Supplementary Methods **2**

Supplementary Figure S1 . . . . **3**

Supplementary Figure S2 . . . . **4**

Supplementary Figure S3 . . . . **5**

Supplementary Table S1 . . . . **6**

Supplementary Table S2 . . . . **7**

Supplementary Methods

Formal definitions

Throughout, let k denote the model / hypothesis, and D our observed data (the time periods between pandemics). When $k = 1$, the data follows an exponential distribution (parameterised by the rate parameter) so that $D_i \sim \text{Exp}(\lambda_1)$, for $i = 1, 2, \ldots, N$. For $k = 2$, the data follows a gamma distribution (parameterised by shape and an inverse mean parameter) so that $D_i \sim \text{Gamma}(\kappa, \lambda_2)$.

Reversible Jump MCMC

Here we describe the two types of moves that the Reversible Jump MCMC (RJMCMC) algorithm [1] consists of; within-model moves and reversible jump moves.

In the within-model moves, each of the relevant parameters λ_1, λ_2, κ were updated using the Metropolis-Hastings algorithm [2, 3], with a Gaussian proposal; for example, $\lambda_1' \sim N(\lambda_1, \sigma')$, with σ' 'tuned' to give an adequate acceptance rate.

Next we describe the reversible jump moves. Firstly, consider the move from model 1 (exponentially distributed model) to model 2 (gamma distributed model). This move requires an auxiliary random variable U. We generated u from a Gamma(α, β) distribution (parameterised by shape and scale parameters). For the uninformed 'not clockwork' and 'weakly mechanistic' κ prior cases, we fixed $\alpha = 2$ and $\beta = 2$. These were altered for the 'strongly mechanistic' κ prior case, with $\alpha = 4$ and $\beta = 1$. These were chosen so as to optimise mixing between the two models. Then set $\kappa = u$ and leave the parameter λ as it is. Note that the reverse move, from model 2 to model 1, requires no auxiliary random variable. Instead, we simply maintain the value of the parameter λ. The Jacobian factor for these model transformations is 1. The acceptance probability for the proposed move from model 1 to 2 is min $\{1, A_{1,2}\}$, where

$$A_{1,2} = \frac{L(D|\lambda,\kappa)p(\lambda,\kappa|k=2)p(k=2)}{L(D|\lambda)p(\lambda|k=1)p(k=1)} \left(\frac{u^{\alpha-1}e^{-\frac{u}{\beta}}}{\beta^{\alpha}\Gamma(\alpha)} \right)^{-1},$$

and from model 2 to 1 is min$\{1, A_{2,1}\}$, where

$$A_{2,1} = \frac{L(D|\lambda)p(\lambda|k=1)p(k=1)}{L(D|\lambda,\kappa)p(\lambda,\kappa|k=2)p(k=2)} \left(\frac{\kappa^{\alpha-1}e^{-\frac{\kappa}{\beta}}}{\beta^{\alpha}\Gamma(\alpha)} \right),$$

with $p(k = i)$ corresponding to the prior assigned to model i. Our priors on each model were $p(k = 1) = p(k = 2) = 0.5$. Note that $A_{1,2}$ and $A_{2,1}$ are reciprocals after change of notation.

Motivation for priors

Supplementary Fig. S1 shows the consequences of different mean-25 gamma distributions for the times between pandemics to help to visualise the consequences of different prior assumptions for the levels of dispersal that are *a priori* credible. In addition, we illustrate the three prior assumptions used for κ to detail the credibility given to differing levels of regularity in each assumption.

Supplementary Figure S1

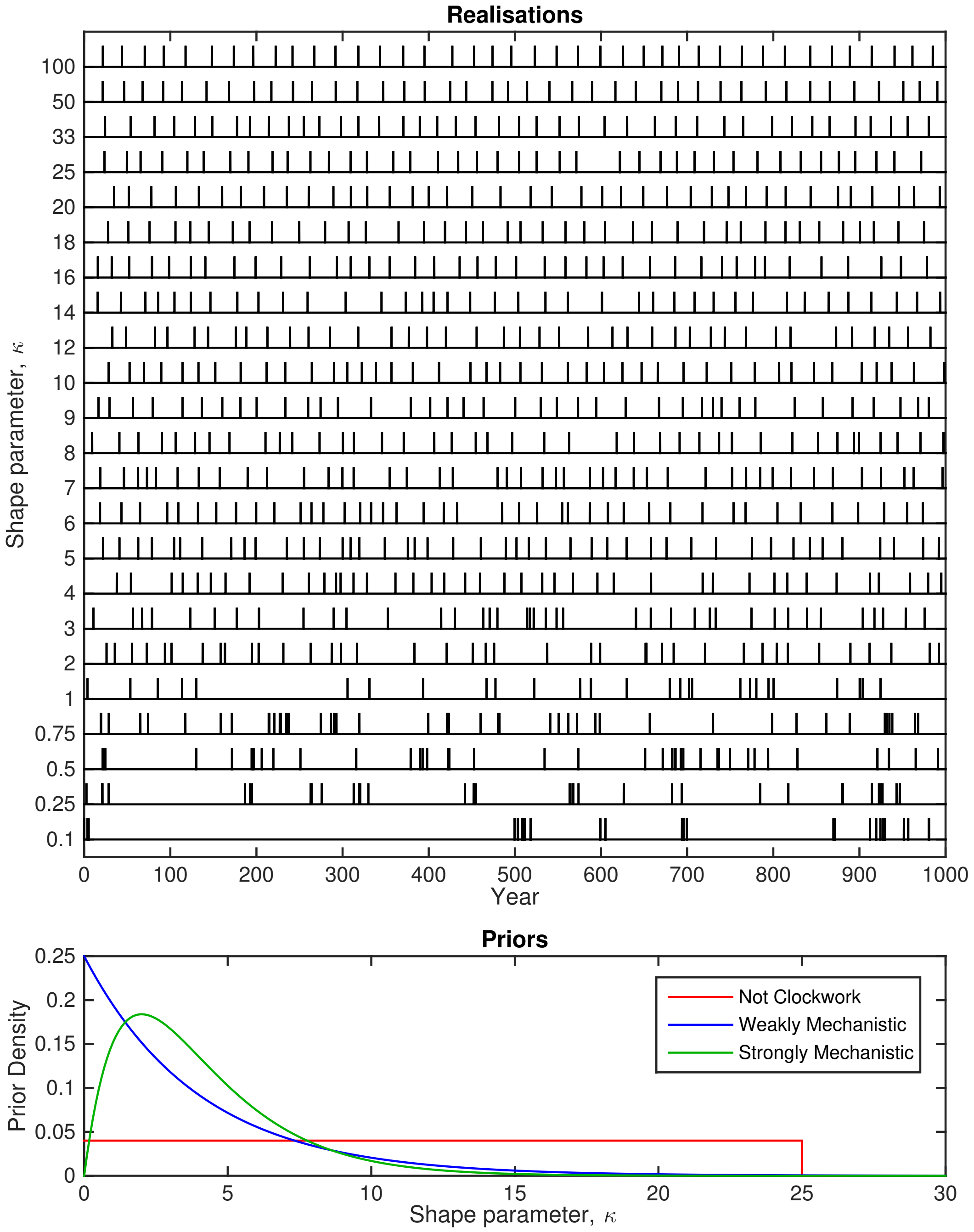

(Top) Visualisation of the implications of different gamma-distributed times (each with mean 25) between pandemics. **(Bottom)** Credibility given by the three κ priors to differing levels of regularity.

Supplementary Figure S2

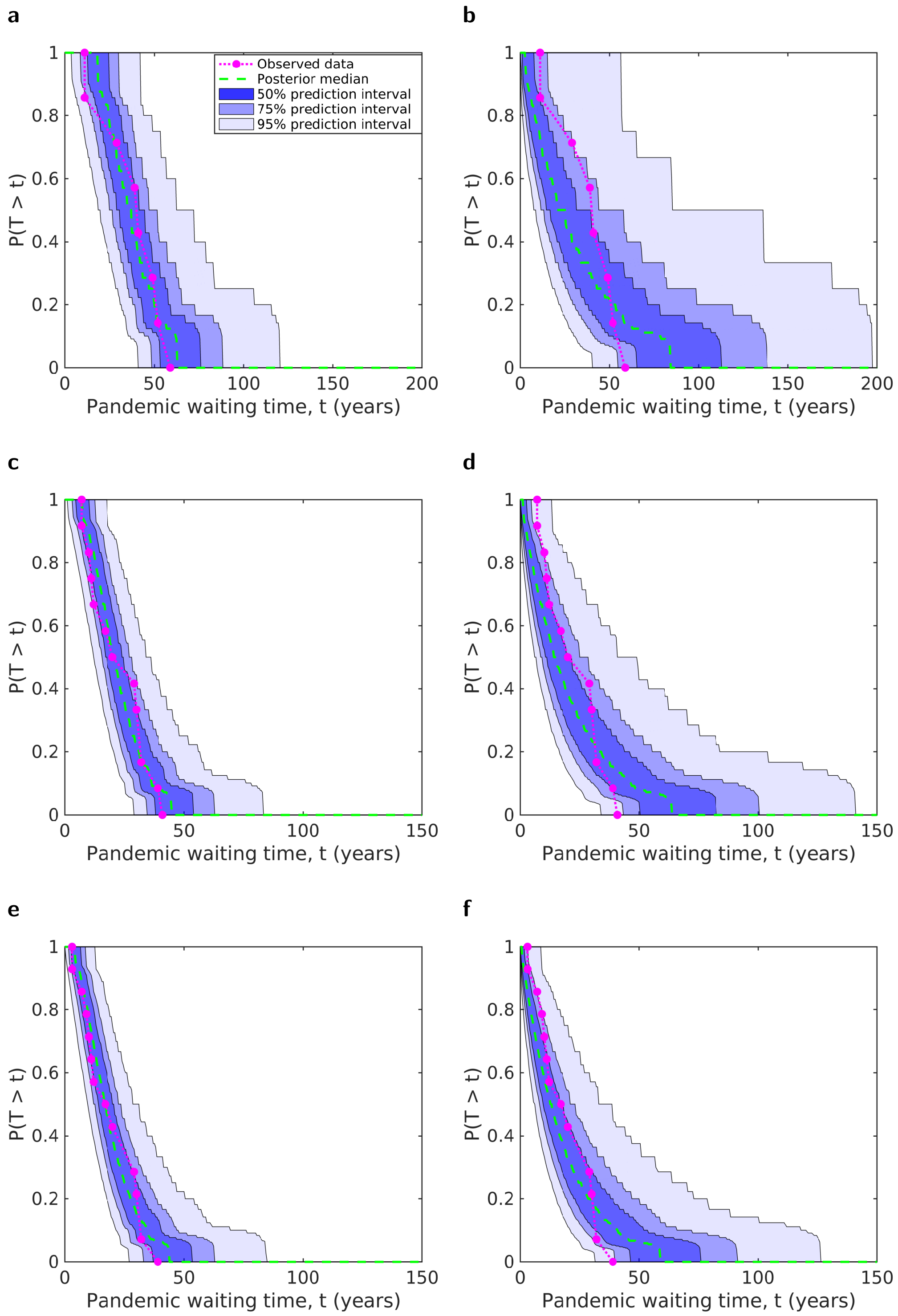

Predicted posterior influenza pandemic inter-event time survival functions versus the empirical survival function, under the 'not clockwork' prior assumption. Waiting time model fits relative to the observed data (magenta dotted line) under the following hypothesis: **(left)** history-dependent; **(right)** memoryless. Across all proposed historic pandemic lists the history-dependent hypothesis corresponds adequately with the observed data. **(a,b)** Timeline A; **(c,d)** timeline B; **(e,f)** timeline C.

Supplementary Figure S3

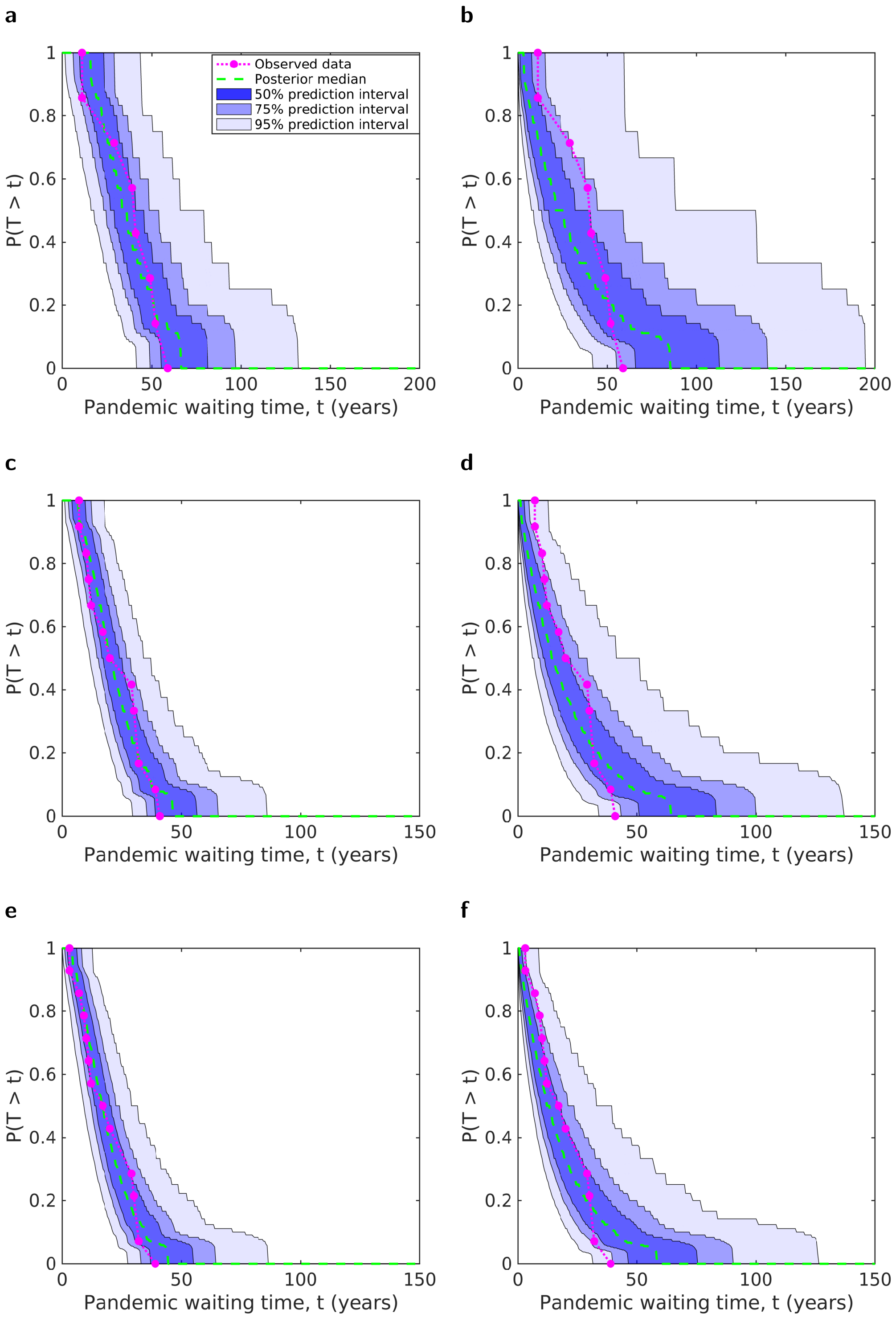

Predicted posterior influenza pandemic inter-event time survival functions versus the empirical survival function, under the 'weakly mechanistic' prior assumption. Waiting time model fits relative to the observed data (magenta dotted line) under the following hypothesis: **(left)** history-dependent; **(right)** memoryless. Across all proposed historic pandemic lists the history-dependent hypothesis corresponds adequately with the observed data. **(a,b)** Timeline A; **(c,d)** timeline B; **(e,f)** timeline C.

Supplementary Table S1

Model Fitting I for each κ prior and timeline.

κ prior	Timeline	Model	Param.	Value	(95% CI)	π
**Not clockwork**	**A**	Exponential	λ_1	0.0273	(0.0122, 0.0515)	0.30
		Gamma	λ_2	0.0255	(0.0175, 0.0362)	0.70
			κ	5.03	(1.61, 11.8)	
	**B**	Exponential	λ_1	0.0453	(0.0247, 0.0751)	0.23
		Gamma	λ_2	0.0435	(0.0315, 0.0588)	0.77
			κ	3.68	(1.58, 7.19)	
	**C**	Exponential	λ_1	0.0522	(0.0300, 0.0837)	0.55
		Gamma	λ_2	0.0510	(0.0357, 0.0707)	0.45
			κ	2.59	(1.21, 4.80)	
**Weakly mechanistic**	**A**	Exponential	λ_1	0.0275	(0.0123, 0.0512)	0.18
		Gamma	λ_2	0.0256	(0.0166, 0.0383)	0.82
			κ	3.81	(1.24, 8.84)	
	**B**	Exponential	λ_1	0.0452	(0.0248, 0.0753)	0.11
		Gamma	λ_2	0.0436	(0.0308, 0.0600)	0.89
			κ	3.23	(1.40, 6.32)	
	**C**	Exponential	λ_1	0.0523	(0.0299, 0.0832)	0.27
		Gamma	λ_2	0.0510	(0.0353, 0.0714)	0.73
			κ	2.40	(1.12, 4.46)	
**Strongly mechanistic**	**A**	Exponential	λ_1	0.0274	(0.0124, 0.0520)	0.14
		Gamma	λ_2	0.0257	(0.0168, 0.0379)	0.86
			κ	3.85	(1.41, 8.30)	
	**B**	Exponential	λ_1	0.0451	(0.0245, 0.0747)	0.082
		Gamma	λ_2	0.0436	(0.0311, 0.0596)	0.92
			κ	3.31	(1.52, 6.22)	
	**C**	Exponential	λ_1	0.0524	(0.0301, 0.0835)	0.23
		Gamma	λ_2	0.0511	(0.0356, 0.0708)	0.77
			κ	2.50	(1.22, 4.50)	

Fitted parameter values and 95% credible intervals for each parameter in the rate / shape parameterisation (to 3.s.f.) and marginal posterior π for each model (to 2.s.f.) in the three timelines considered.

Supplementary Table S2

Model Fitting II for each κ prior and timeline.

κ prior	Timeline	Model	Param.	Value	(95% CI)	π
**Not clockwork**	**A**	Exponential	μ_1	36.6	(19.4, 81.8)	0.30
		Gamma	μ_2	39.3	(27.6, 57.1)	0.70
			σ	17.4	(10.8, 35.7)	
	**B**	Exponential	μ_1	22.1	(13.3, 40.5)	0.23
		Gamma	μ_2	23.0	(17.0, 31.8)	0.77
			σ	11.9	(7.99, 20.9)	
	**C**	Exponential	μ_1	19.1	(12.0, 33.4)	0.55
		Gamma	μ_2	19.6	(14.1, 28.0)	0.45
			σ	12.1	(8.18, 21.2)	
**Weakly mechanistic**	**A**	Exponential	μ_1	36.3	(19.5, 81.3)	0.18
		Gamma	μ_2	39.0	(26.1, 60.2)	0.82
			σ	19.7	(12.2, 41.8)	
	**B**	Exponential	μ_1	22.1	(13.3, 40.4)	0.11
		Gamma	μ_2	22.9	(16.7, 32.4)	0.89
			σ	12.7	(8.44, 22.5)	
	**C**	Exponential	μ_1	19.1	(12.0, 33.4)	0.27
		Gamma	μ_2	19.6	(14.0,28.3)	0.73
			σ	12.6	(8.40, 22.1)	
**Strongly mechanistic**	**A**	Exponential	μ_1	36.5	(19.2, 80.8)	0.14
		Gamma	μ_2	39.0	(26.4, 59.4)	0.86
			σ	19.6	(12.4, 39.5)	
	**B**	Exponential	μ_1	22.2	(13.4, 40.8)	0.082
		Gamma	μ_2	22.9	(16.8, 32.2)	0.92
			σ	12.5	(8.48, 21.6)	
	**C**	Exponential	μ_1	19.1	(12.0, 33.2)	0.23
		Gamma	μ_2	19.6	(14.1, 28.1)	0.77
			σ	12.3	(8.36, 21.1)	

Fitted parameter values and 95% credible intervals for each parameter in the mean / standard deviation parameterisation (to 3 s.f.) and marginal posterior π for each model (to 2.s.f.) in the three timelines considered.

References

[1] P. J. Green. Reversible jump Markov chain Monte Carlo computation and Bayesian model determination. *Biometrika*, **82**(4):711–732, 1995. doi: 10.1093/biomet/82.4.711.

[2] N. Metropolis, A. W. Rosenbluth, M. N. Rosenbluth, A. H. Teller, and E. Teller. Equation of State Calculations by Fast Computing Machines. *The Journal of Chemical Physics*, **21**(6):1087–1092, 1953. doi: 10.1063/1.1699114.

[3] W. K. Hastings. Monte Carlo Sampling Methods Using Markov Chains and Their Applications. *Biometrika*, **57**:97–109, 1970. doi: 10.1093/biomet/57.1.97.
